# The development and applications of circulating tumour cells, circulating tumour DNA and other emerging biomarkers for early cancer detection

**DOI:** 10.37349/etat.2025.1002314

**Published:** 2025-05-13

**Authors:** David Sinclair Thomas Junior, Junjie Chai, Yong-Jie Lu

**Affiliations:** IRCCS Istituto Romagnolo per lo Studio dei Tumori (IRST) “Dino Amadori”, Italy; ^1^Barts and the London School of Medicine and Dentistry, Queen Mary University of London, E1 2AD London, UK; ^2^Centre for Biomarkers and Therapeutics, Barts Cancer Institute, Barts and the London School of Medicine and Dentistry, Queen Mary University of London, EC1M 6BQ London, UK

**Keywords:** Early cancer detection, cancer biomarkers, liquid biopsy, circulating tumour cells (CTCs), circulating tumour DNA (ctDNA)

## Abstract

Despite major improvements in cancer treatment, detection, and health promotion, the mortality rates of late-stage cancer remain high. This is a critical issue because a large proportion of cancer mortality is experienced by patients who have late-stage disease at diagnosis. As survival is substantially higher for almost all cancers when diagnosed at an early stage, effective early cancer detection strategies could drastically reduce overall cancer mortality. Advances in various technologies have culminated in the development of liquid biopsies. The tumour biomarkers applied for non- or minimally-invasive cancer detection include tumour cells and their components in bodily fluids, especially peripheral blood for circulating tumour biomarkers. The most well-studied circulating tumour biomarkers in recent years for the early detection of cancer are circulating tumour cells (CTCs) and circulating tumour DNA (ctDNA), with research into other classes rapidly expanding. CTCs and ctDNA have been detected at an early stage in several types of cancer with high specificity, aiding risk stratification and, in some cases, identifying clinically actionable molecular features. Therefore, these circulating biomarkers offer several advantages over the traditional cancer detection methods. Although their limitations are considerable, the evolving evidence suggests they have tremendous potential as tools for early cancer detection. In this review, we evaluate the development and applications of circulating biomarkers for early cancer detection, with a focus on CTCs and ctDNA. We also briefly explore the emerging evidence on extracellular vesicles, circulating proteins and synthetic biomarkers, discuss the limitations of current approaches and provide suggestions to achieve further progress in this setting.

## Introduction

Cancer is a public health issue of global importance. An estimated 10 million people died of cancer in 2020 [1], accounting for one in six deaths worldwide [2]. This statistic serves as a stark reminder that cancer is a leading cause of mortality, despite notable improvements in cancer-related outcomes over recent decades. In the UK, net five-year survival for all cancers combined has doubled, rising to 54% since 1971 [3], while overall mortality has fallen by 19% [4]. Similar progress has occurred in many other countries, which is widely attributed to reduced smoking rates, breakthroughs in cancer treatment and advances in cancer detection [5, 6]. Although these achievements have transformed many lives, the mortality rates for late-stage cancer remain high [1]. The incidence of cancer is also expected to reach 28 million globally by 2040 [1], with rising incidence and mortality rates for people aged under 50 on the horizon [6]. Thus, strategies to further improve cancer-related outcomes are urgently needed.

Facilitating the early detection of cancer has enormous potential to improve patient outcomes. Survival is substantially higher for nearly all types of cancer when detected at an early stage [7]. For example, the five-year survival rate of colorectal cancer when diagnosed at stage I exceeds 90% but falls to ~13% at stage IV in several countries [7, 8]. This discrepancy is important because in the US, cancer first diagnosed at stage IV is estimated to account for 18% of diagnoses but 45% of all cancer deaths in patients aged 50–79 [9], suggesting late-stage disease at the point of diagnosis forms a large percentage of cancer mortality. The issue of late diagnosis is further highlighted by the increased proportion of stage III/IV cancers due to diagnostic delays during the COVID-19 pandemic [10], contributing to the observed excess mortality [11]. In England, almost half of all cancers were diagnosed at these late stages before the pandemic [12]. Hence, strategies to detect these cancers at earlier, more treatable stages could significantly reduce the overall burden of cancer-related mortality.

Histopathology is the standard for cancer diagnosis due to its high accuracy. However, this approach requires a biopsy or surgical resection of the tumour. These invasive procedures are not appropriate for all patients because of their risks, require a sufficient tumour volume, and typically, in the form of imaging, need clinical evidence of cancer to be done [13]. Thus, a rate-limiting step for achieving a diagnosis with histopathology is the initial detection of the cancer, restraining its use for early diagnosis. Tumour heterogeneity further complicates matters, as the subpopulations of cancer cells within a tumour vary in their pathobiology and molecular features [14, 15]. Consequently, the obtained grade and molecular profile only represent the exact part of the tumour sampled at that timepoint, especially since biopsies are seldom repeated. As there is no standardised guidance to address biases from the sampling process of biopsies or tumour heterogeneity [15], the current methods of tissue analysis hinder the accurate assessment of every patient’s cancer at diagnosis.

Imaging has a few limitations in the context of early cancer detection. Due to the level of background activity under clinical conditions, even sensitive modalities like positron emission tomography-computed tomography (PET-CT) cannot reliably detect tumours smaller than 0.5 cm^3^, hindering the diagnosis of low volume, early-stage cancers [16]. The standard, widely available imaging methods are also prone to false-positive (FP) results. Over half of all women screened annually for breast cancer with mammography will have at least one FP over 10 years [17]. Similarly, lung cancer screening with low-dose CT has a baseline FP rate of 9.6–28.9%, despite only being available to high-risk patients [18]. Moreover, the overdiagnosis of indolent, slow-growing tumours that are unlikely to cause symptoms or reduce life expectancy is a key challenge for imaging-based detection methods. Around 25% of breast and 13–25% of lung cancers detected by screening are overdiagnosed [19]. Although screening with mammography and low-dose CT is estimated to reduce breast and lung cancer mortality by 19% [17] and 15–20%, respectively [18, 19], these reductions are partly offset by the harms of FP results and the overdiagnosis of indolent tumours. These include complications from further investigation and overtreatment, as well as emotional distress [19]. Hence, there is an urgent need for methods of cancer detection that, whether used alone or in combination with existing methods, are accurate, have low FP rates and distinguish aggressive, clinically relevant cancers from indolent ones.

To surpass the limits of current cancer detection methods, research into alternative technologies accelerated over the last few decades, resulting in the development of the liquid biopsy. This term covers a range of assays that offer a non- or minimally-invasive, easily repeated way to detect cancer cells and/or their components in biofluids [20, 21]. Circulating tumour biomarkers (CTBs), especially those in peripheral blood, are among the commonly applied tumour biomarkers in cancer liquid biopsies. Early research on CTBs focussed on detecting tumour-associated serum and plasma proteins. Some of these biomarkers, such as carcinoembryonic antigen (CEA), cancer antigen-125 (CA-125), and prostate-specific antigen (PSA), remain in clinical use. These CTBs are still routinely used to assess cancer prognosis, treatment response and disease recurrence [22], but due to the limited sensitivity and specificity of these circulating protein biomarkers, their role in early cancer detection is restricted.

Respectively, less than 25% and 50% of all early-stage colorectal and ovarian cancers express CEA and CA-125, resulting in low sensitivities [23]. Factors other than cancer can also affect their expression, imparting a high risk of FP results. For instance, endometriosis and pregnancy increase serum CA-125 levels, while age and benign prostatic hypertrophy increase PSA [23]. Thus, these biomarkers lack the sensitivity and specificity needed to accurately detect early-stage cancer, especially in asymptomatic patients. The issues with these biomarkers are showcased by the results of a European multinational randomised-controlled trial (RCT) that used serum PSA levels to screen for prostate cancer in average-risk populations [19, 24]. A small number of men benefited; one death was prevented for every 780 men screened using a threshold of ≥ 3–4 ng/mL. However, there was a high rate of FP results, especially in the first round of screening, and many of the PSA-detected cancers were overdiagnosed. Due to the widespread harm that would occur, these biomarkers are therefore not recommended for population-level screening. Excitingly, novel CTBs have materialised in recent years that may overcome the limitations of traditional CTBs and other conventional methods in the pursuit of early cancer detection.

**Figure 1:**
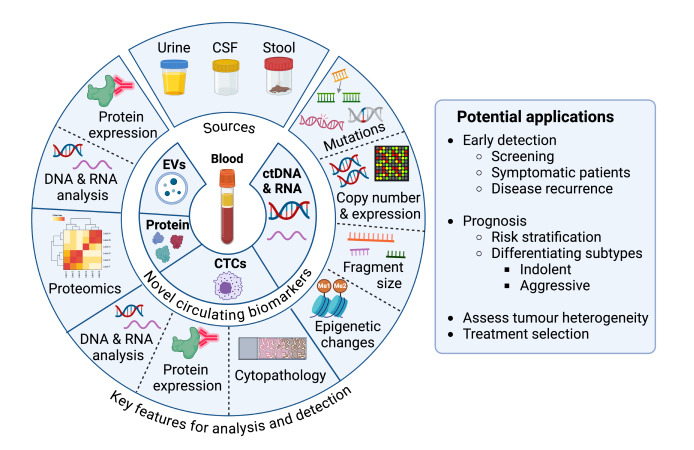
**Novel circulating tumour biomarkers: main types, sources, features utilised for detection and potential applications in early cancer detection.** The figure outlines the most common types of novel circulating cancer biomarkers, the biological fluids frequently used as sources, the key features that are typically exploited for analysis or detection, and their potential applications in early cancer detection. Created in BioRender. Lu, Y. (2025) https://BioRender.com/r83g977. Adapted from (Herath et al., 2022) [21], © 2022 Herath, Sadeghi Rad, Radfar, Ladwa, Warkiani, O’Byrne and Kulasinghe, and consolidated with other sources: see bibliography [22, 27–30]. CTCs: circulating tumour cells; ctDNA: circulating tumour DNA; CSF: cerebrospinal fluid; Me1: mono-methylation; Me2: di-methylation; EVs: extracellular vesicles

With the unprecedented advance of research technologies, other circulating tumour materials, such as tumour cells, nucleic acids, and extracellular vesicles (EVs), have been extensively investigated in the last two decades for their potential as cancer biomarkers, and they may revolutionise early cancer detection [25, 26]. As shown in Figure 1, the most well-studied CTBs in recent years include circulating tumour cells (CTCs) and circulating tumour DNA (ctDNA), although interest in circulating EVs, novel protein combinations, tumour metabolites, and other classes of CTBs is increasing [27–31]. Until recently, most studies on CTBs focussed on their utility in prognostication, disease monitoring and treatment selection for late-stage cancer. However, the emerging evidence suggests these CTBs are present at an early stage in some cancers, are prognostic, and have high specificity relative to the existing detection methods [27–31]. CTCs and ctDNA may also accurately account for tumour heterogeneity [14], providing molecular profiles that avoid biases associated with tissue biopsies and detect clinically actionable molecular features. In addition, these CTBs may potentially help to differentiate more aggressive, clinically relevant cancers from indolent ones [27–30]. Herein, we intend to provide an up-to-date analysis on the development and applications of CTCs and ctDNA, which have been assessed across a range of cancer types, while briefly examining a few other emerging CTBs in the context of early cancer detection.

## CTCs

### An introduction to CTCs

CTCs are cancer cells that have entered the circulatory system after detaching from a tumour and are likely intermediate components of the metastatic cascade. The spread of cancer cells from the primary tumour to distal sites involves a sequence of key events [27]. After acquiring alterations that underpin the hallmarks of cancer [32], the cells destined to metastasise must detach from the tumour. This initial step can be achieved through epithelial-mesenchymal transition (EMT), a process whereby epithelial cancer cells lose some of their properties, such as intercellular adhesion, while gaining mesenchymal traits that promote resistance to anoikis and enhance the invasion of local blood and lymphatic vessels [33]. The cells must then enter the circulation, probably via active migration and invasion, passive shedding or a combination of such processes [34]. Upon intravasation, these cells become CTCs, which must endure hydrodynamic shear forces, avoid clearance by immune cells and, ultimately, extravasate into distal tissues. If successful, they become the disseminated tumour cells (DTCs) that can establish dormant DTC deposits and, under certain conditions, active metastatic lesions [35]. The few CTCs that successfully overcome these significant obstacles may therefore play a crucial role in metastasis; an assertion supported by multiple lines of evidence.

In immunodeficient mice, orthotopic injection of human breast cancer cells causes the formation of primary tumours, endogenous CTCs, and lung metastases derived from CTCs [36]. Similarly, intraosseous injection of patient-derived breast CTCs causes bone, lung, and liver metastasis [37], while injection of colorectal CTCs into the spleen triggers liver metastasis [38]. Thus, CTCs likely can seed metastases. However, the ability to metastasise is not equal across all CTCs, with some subtypes having a greater seeding capacity than others. In some models, cancer cells with a hybrid EMT phenotype form more tumour spheres in vitro and metastasise more efficiently in vivo than fully epithelial and mesenchymal cells [39]. Correspondingly, CTCs with a hybrid EMT phenotype could efficiently form metastases in a few xenograft models [37, 39]. Such behaviours mirror those associated with cancer stem cells, which are the subset of tumour cells considered to be capable of the extensive proliferation needed for initiating metastatic lesion formation [38, 40]. In addition, homotypic CTC clusters in the former study had 50-fold higher rates of metastasis than single CTCs [36], agreeing with results from other studies [41]. In a different xenograft model, heterotypic CTC clusters containing neutrophils metastasised significantly faster than single CTCs, and their presence correlated to shorter survival [42]. Moreover, CTC-platelet clusters seem less prone to shear stress and detection by natural killer cells, potentially contributing to the observed reduction of metastasis in mice with non-functional or absent platelets [43]. Such results are consistent with the growing reports of these subtypes having prognostic value in various late-stage cancers [27, 33, 35], in some cases predicting worse overall or progression-free survival than single CTCs [41, 42]. Overall, these findings indicate that subsets of the CTC repertoire are not just byproducts of the cascade and can play direct roles in metastasis, whether individually or by working together. Furthermore, multiple lines of evidence suggest the cascade begins in the early stages of cancer [27]. As metastasis is the cause of most cancer-related mortality, the roles of CTCs in this process have clear implications on their use as a tool for early cancer detection.

Over the last 20 years, numerous studies have shown that cancer cell dissemination can occur at the earliest stages of disease. In contrast to the traditional models of cancer progression, CTCs could seed the liver before primary tumours were histologically detected in a mouse model of pancreatic adenocarcinoma [44]. Further supporting this finding, DTCs have been detected in the bone and CTCs in the peripheral blood of patients with various early-stage cancers [27], including (for CTCs) breast and colorectal cancer [45, 46]. DTCs have also been identified in the bone of patients with ductal adenocarcinoma in situ [47], indicating that CTCs are released during the pre-cancerous stage of disease in some patients. This is reinforced by the detection of CTCs in patients with colorectal adenomas [48]. Together, these results suggest that the presence of CTCs in early-stage cancer might indicate that the first steps of the metastatic cascade have been initiated. Moreover, CTCs can be prognostic in some early-stage cancers, including those mentioned [45, 46]. Hence, biological differences may exist in cancer patients who have CTCs at an early stage that contribute to their worse outcomes [27]. CTC-based assays could therefore potentially help to identify patients with more aggressive disease earlier.

### The development of technologies for CTC isolation and detection

**Figure 2:**
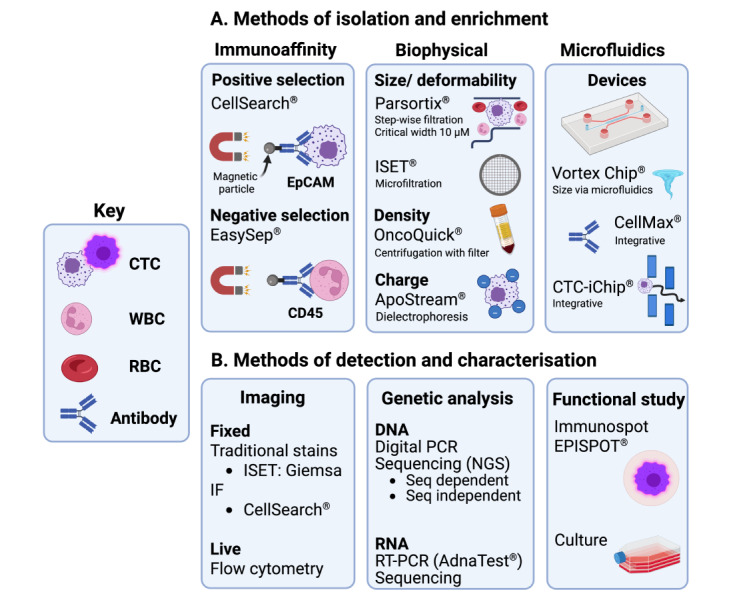
**The methods of isolation and detection of CTCs.** (**A**) The commonest methods of CTC isolation. The current methods of isolation mostly exploit the presence of cell surface proteins on CTCs or other cell types with antibodies, differences in the physical properties of CTCs, or how they interact with microfluid dynamics. True microfluidic devices use microfluid dynamics to isolate CTCs, which mainly relies on the size of CTCs (e.g., Vortex/VTX-1 Chip), though some devices use one or more additional methods to further improve yield, such as immunoaffinity (e.g., CellMax); (**B**) the methods of CTC detection. Several methods are used to detect CTCs, including IF, PCR, sequencing and functional study. Created in BioRender. Lu, Y. (2025) https://BioRender.com/x22g161. Adapted from (Lone et al., 2022) [25], © The Author(s) 2022, and consolidated with other sources: see bibliography [35, 53, 55, 57, 62]. CD45: cluster of differentiation 45; CTCs: circulating tumour cells; EpCAM: epithelial cell adhesion molecule; EPISPOT: EPithelial ImmunoSPOT; IF: immunofluorescence; ISET: isolation by size of epithelial cells; NGS: next generation sequencing; PCR: polymerase chain reaction; RT-PCR: reverse transcriptase-PCR; RBC: red blood cell; Seq: sequencing; WBC: white blood cell

Although CTCs were first reported over 150 years ago [49], the advances in technology that have allowed their isolation, characterization, and evaluation as a cancer biomarker only occurred in recent decades. Numerous factors complicate the isolation of CTCs from peripheral blood. Relative to the billions of red (RBC) and white (WBC) blood cells, CTCs are rare, with the mentioned subtypes rarer still. Reported CTC counts even in metastatic patients are typically around 5–50 CTCs/7.5 mL of peripheral blood but vary widely between patients due to several factors [50]. As most CTCs die in the hostile intravascular space, CTCs only have an estimated half-life of minutes to hours [36]. Their sizes, shapes, expression of cell surface markers and genetic profiles are also heterogeneous, even in the same patient [35]. As summarised in Figure 2, a diverse range of assays have been developed that exploit these features to selectively capture and detect CTCs. By integrating multiple enrichment methods, microfluidic platforms are an emerging method for the efficient detection of CTCs. Further progress will likely allow CTC-based assays to complement or improve upon the existing techniques for early cancer detection.

Immunoaffinity is one of the most widely used technologies in CTC isolation and detection assays. CellSearch was the first clinically approved method for CTC detection by the FDA, which established the prognostic value of CTC counts in metastatic breast, prostate and colorectal cancer [51]. This assay uses antibody-conjugated ferroparticles (Figure 2A) that bind with high affinity to CTCs expressing epithelial cell adhesion molecule (EpCAM), a cell-surface protein present in most carcinomas, allowing their isolation with magnets. Positively selected CTCs are then visualised (Figure 2B) with immunofluorescence (IF), using fluorescent antibodies against EpCAM, epithelial cytokeratins, and CD45 to exclude FPs from WBCs [52].

Most immunoaffinity-based assays isolate CTCs by targeting EpCAM. However, some assays use negative selection to enrich for CTCs instead, usually by depleting WBCs with anti-CD45 antibodies (Figure 2A) [52, 53]. The detection method depends on the assay. Some combine isolation and detection on a single platform, like CellSearch, while others have a separate detection step. For example, the AdnaTest assay detects CTC RNA after isolation with EpCAM antibodies [53], but other detection methods, such as sequencing, could be used instead (Figure 2B). Overall, immunoaffinity assays have high specificity [52] and, with CellSearch, allow the ‘direct’ evaluation of CTCs. Assessing the expression of tissue-specific proteins on CTCs, as well as the presence of specific genomic alterations, can also reveal the likely origin of the primary tumour. On the other hand, positive selection misses CTCs that express EpCAM at low levels or are EpCAM-negative (EpCAM^neg^), like those that undergo EMT [33, 52]. These issues particularly reduce sensitivity and increase the risk of false-negatives (FNs) in early-stage cancer because CTC counts broadly correlate to disease stage [51]. FP results from non-cancerous EpCAM-positive (EpCAM^pos^) cells also occur in 2–5% of those tested with CellSearch who are healthy or have non-malignant disease [54]. Moreover, the bulk removal of WBCs with negative selection predisposes the loss of CTCs in clusters [53]. Without further advances in how this technology is applied, these issues limit the potential of immunoaffinity assays for early detection.

The physical properties of cells can also be used to isolate CTCs from peripheral blood. The OncoQuick method uses centrifugation to separate high-density RBCs and WBCs from CTCs in density-gradient medium (Figure 2A), while the integrated membrane traps CTCs and allows lower-density material to flow through [55]. Methods like OncoQuick have clear advantages for CTC isolation compared to immunoaffinity. Besides high recovery rates (70–90% for OncoQuick [55]), EpCAM^neg^ CTCs can be isolated by size and density-based assays. These methods also generally retain CTC viability, increasing the number and quality available for detection or functional study [53] (Figure 2B). The strengths of biophysical separation methods are further highlighted by the second FDA-approved CTC isolation device, Parsortix. The device isolates CTCs by trapping them in progressively narrowing channels (Figure 2A), due to their larger size and lower deformability [56]. We have demonstrated that Parsortix not only efficiently isolates CTCs with epithelial features, but also cytokeratin-negative mesenchymal CTCs at higher numbers than epithelial CTCs with high purity and viability [57]. In a study on prostate cancer, we further demonstrated that CTCs isolated with Parsortix could be valuable for the early detection of clinically significant prostate cancer [58]. However, these size-based approaches may lose small CTCs (< 5 μm) [53], which can have prognostic value, as recently shown in a small cohort of early-stage non-small cell lung cancer (NSCLC) patients [59].

Recent work has focussed on developing microfluidic devices that use novel, integrative technology to isolate CTCs more efficiently, promising major improvements over the initial assays for CTC isolation and detection. Microfluidic devices largely utilise microscale fluid dynamics to isolate CTCs based on their biophysical differences. Depending on the device, CTCs are then detected using on- or off-platform (Figure 2B) techniques [60]. For example, the Vortex (Figure 2A) device generates microvortices that trap CTCs due to their larger size and lower density [60]. In a pilot study, this device isolated CTCs above the healthy patient cutoff (1.25 CTCs/mL blood) in 85% of the 13 metastatic breast and lung cancer patients, whereas CellSearch only managed 15% [61]. In addition, the ApoStream device exploits the higher zeta potential of CTCs to separate them through electrophoresis (Figure 2A) under microscale laminar flow conditions [60]. In one study, ApoStream isolated CTCs from all nine late-stage NSCLC patients (4/9 had hundreds), while CellSearch isolated between 1–8 CTCs in only three patients [62]. As both devices also capture EpCAM^neg^ and mesenchymal CTCs [61, 62], these studies highlight the advantages of label-free microfluidic platforms relative to CellSearch.

In contrast, the 3rd generation CTC-iChip improved on CellSearch by combining the strengths of microfluidic and immunoaffinity-based technology. The device works by displacing CTCs and WBCs from RBCs and platelets as they collide in a size-dependent manner with microposts (Figure 2A). The CTCs are then precisely aligned by inertial forces as they pass the curves of the main channel, allowing their capture with EpCAM antibodies or enrichment via WBC depletion [60, 63]. Showcasing its higher sensitivity, the EpCAM-iChip isolated significantly more CTCs than CellSearch in 61% (22/36) of metastatic prostate cancer patients with low counts (< 25/7.5 mL) [63]. Such integrative devices hold much promise for efficient CTC capture and single cell analysis.

Most of these studies have several key limitations, including their small sample sizes, lack of consistent reporting for preanalytical factors like whether patients had surgery or treatment before blood draws, and inclusion of mainly late-stage patients. However, the emerging clinical evidence indicates the potential utility of some devices in early cancer detection.

### The applications of CTCs in early cancer detection

An early prospective study used CellSearch to assess CTC positivity at diagnosis and its prognostic value for 602 breast cancer patients [64]. Most of the cohort had stage I-II disease (87.3%), but only 16.9% of these patients had at least 1 CTC/mL of blood, rising to 31.4% for stage III disease. These results agree with other CellSearch-based studies that found typical positivity rates of 11–24% in early-stage breast cancer, which increase at more advanced disease stages [27, 65]. Importantly, having ≥ 1 CTC in this study was an independent risk factor for death, suggesting the subset of early-stage breast cancer patients with detectable EpCAM^pos^ CTCs could have more aggressive disease. These patients might therefore benefit from the use of CTC-based assays to detect their cancer earlier and aid risk stratification. Several other studies have reported that CTC counts predict overall and distant disease-free survival in early breast cancer, while some studies did not replicate these findings [65]. Due to the inability of CellSearch to detect EpCAM^neg^ CTCs, the reported CTC positivity rates and counts in these early-stage patients are probably underestimates [52]. This assertion is supported by recent studies that have used integrative microfluidic devices to detect CTCs, such as CytoSorter, which achieved a positivity rate of > 86% in a group of 105 patients known to have stage I-II breast cancer [66].

Another early study assessed CTC positivity and its prognostic value in a cohort of 94 colorectal cancer patients [67]. In this cohort, 29.4% of stage I-II patients had ≥ 2 CTCs detected by CellSearch, with positivity correlating to disease stage. However, CTC positivity was not prognostic in this study. Yet, a meta-analysis of studies using various methods of detection, including CellSearch, identified CTCs as an independent marker of poorer survival for non-metastatic colorectal cancer patients [68]. This inconsistency likely reflects differences in cohort size, the variable performance of CellSearch across studies, and preanalytical factors that influenced the average CTC counts obtained, similarly to the mentioned studies on early breast cancer. Moreover, a recent, large prospective study using the EpCAM-CellMax device had a sensitivity and specificity of > 89% and 84.7% across all stages of colorectal cancer with an area under the receiving operator characteristics curve (AUC) of 0.940. In addition, the device could detect patients with adenomas at an overall sensitivity of 79.2%, and CTC counts could distinguish patients who had adenomas from those with colorectal cancer [69]. Together, these findings suggest that further improvements to study design and the technology underpinning CTC assays could reduce the impact of external factors, enabling the true clinical utility of CTCs in early cancer detection to be more effectively assessed. Further research into their detection performance for patients who have precancerous lesions would be especially useful, due to the lack of investigation in this area.

In prostate cancer, we detected CTCs using the Parsortix system in 63% of a small cohort of 38 treatment-naive localised prostate cancer patients [70]. In the same study, the detection of CTCs with both epithelial and mesenchymal markers was associated with high-risk localised disease and metastasis. For prostate cancer, the goal is to detect clinically significant disease, as detecting all cases leads to overdiagnosis, a major issue with current tools like PSA. The findings of this study suggest that CTCs have potential for the early detection and risk stratification of more aggressive, clinically relevant prostate cancer. This is supported by another study we conducted on CTCs in a larger cohort of 155 treatment-naive localised prostate cancer patients and 98 pre-biopsy patients with concerning PSA levels and/or abnormal examination findings [58]. In this cohort, we found the combination of CTC positivity, CTC gene expression and PSA was highly accurate at predicting biopsy outcomes of clinically significant cancer in the pre-biopsy patients, with an AUC = 0.927.

For pancreatic adenocarcinoma, the EpCAM-NanoVelcro device could detect ≥ 1 CTC in 66.7% (28/42) of patients with stage II-III disease. Setting a threshold of ≥ 3 CTCs distinguished local/regional from metastatic cases in this cohort, with a sensitivity and specificity of 75% and 96.3% (AUC = 0.867) across all stages [27, 71]. Taken together, these studies show that CTCs are present in many types of early-stage cancer, are relatively specific, can help identify more aggressive disease, and have potential as tools for the early detection of clinically relevant cancer and even precancerous lesions.

However, these encouraging results must be interpreted cautiously. All these studies enrolled people who were either symptomatic or known to have cancer. Independent replication of their results in larger or average-risk cohorts has also not been achieved, although RCTs of CTC-based assays for early breast and colorectal cancer detection are ongoing [27]. This is especially relevant for the CellMax study, due to its high-risk cohort. The studies also used different blood volumes (range: 4–30 mL [64, 71]) for isolation as well as varying thresholds or markers to define CTC-positive patients, so direct comparisons between studies are difficult. In addition, the reported sensitivities for early-stage cancer would not be sufficient if screening average-risk populations, as they would be susceptible to FN results. Thus, further improvements in the technology underpinning these assays and how they are used are needed to maximise their utility in early cancer detection strategies.

### Future directions for CTCs in early cancer detection

At present, there are no standardised criteria to outline what morphologic features or cell-surface markers define CTCs [52]. This is important because the ‘direct’ detection of CTCs by IF or other staining methods (Figure 2B) relies on the criteria used to identify them. Although CellSearch was the first FDA-approved CTC detection system, it cannot capture EpCAM^neg^ mesenchymal CTCs, which are increasingly associated with worse outcomes [33, 39]. Such limitations severely hinder the use of CTCs in early detection. Therefore, new standards for the physical features and cell-surface markers should be developed, at least for the validation of new detection methods.

Traditionally, enriched CTCs are manually identified and counted under the microscope regardless of the enrichment method, which is labour-intensive and risks missing CTCs, as they are still vastly outnumbered by WBCs after CTC isolation. Automated, AI-based image analysis has significant advantages in CTC research to increase throughput, consistency, and sensitivity [72, 73]. By leveraging AI algorithms, researchers can rapidly scan complex blood sample images and pinpoint CTCs among the numerous WBCs, reducing the workload, the risk of missing cells from manual review, and the influence of subjective bias from interobserver variability.

Most established CTC assays use IF labelling to distinguish CTCs from WBCs. AI-driven analysis of multi-channel fluorescence images has achieved high accuracy in identifying labelled CTCs. Various machine learning (ML) approaches trained with pre-selected features have been applied in CTC image analysis, including random forest [74], K-nearest neighbours algorithm [75], Bayesian classifier [76], and support vector machine (SVM) [77]. Deep learning (DL), a subset of ML, autonomously extracts features and eliminates biases brought by subjectively pre-selected features, enhancing the accuracy and objectivity of detection. For instance, Zeune and colleagues [72] developed an autoencoder conventional neural network (CNN) model categorizing cell images into five different classes (CTCs, EVs, WBCs, bare nucleus, and unknown objects) with an overall accuracy of 83.8% and an agreement of 65.8% to manual count on CTCs tested with patient samples. Guo et al. [78] developed a novel CNN model using patient samples to detect CTCs based on IF in situ hybridization (imFISH) images with a sensitivity and specificity of 97.2% and 94.0%, respectively. Additionally, Park et al. [79] have explored the application of a hybrid CNN-SVM algorithm for CTC clusters using artificial CTC clusters created by microfluidics containing 2–4 cells per cluster, with a sensitivity and specificity over 90%, demonstrating the potential of AI algorithms in identifying CTC clusters, albeit using relatively uniform cluster images with less heterogeneity compared to clinical scenarios. Nonetheless, these studies were limited to the enumeration of CTCs or CTC clusters and did not address their biological classification into epithelial, mesenchymal, or EMT phenotypes. In light of this gap, we are developing a DL model capable of detecting CTCs and CTC clusters while classifying single CTCs and CTCs within clusters into distinct biological phenotypes. In summary, AI-based automated image analysis is becoming indispensable in CTC research, enhancing our ability to detect these rare cells, although the generalisability of the models requires further validation on patient samples.

Research into alternative cell-surface proteins has achieved promising initial results for early detection, indicating that further work is needed to confirm the most relevant markers for each type of cancer. In a recent prospective study, IF was used to detect CTCs in breast cancer patients by targeting pancytokeratin (PanCK), GATA-3, EpCAM and gross-cystic disease fluid protein-15. This study reported a median sensitivity of 95.8% for patients with stage I-II disease and distinguished people who were healthy or had benign breast disease with a specificity of 93.1% [80]. In addition, another prospective study used PanCK, EpCAM, prostate membrane-specific antigen and alpha methyl-acyl coenzyme-A racemase to detect early-stage prostate cancer. This combination had a sensitivity of 75.0% and specificity of 96.9% for localised prostate cancer and could differentiate it from benign prostate disease [81]. These studies suggest that combining non-specific but sensitive cytokeratins with tissue-specific markers may strike an optimal balance between sensitivity and specificity, thereby improving the accuracy of CTC-based methods for early-stage cancer detection.

Confirming the most clinically relevant features and subtypes of CTCs could improve the detection and risk stratification of early-stage cancer patients. To date, it is unclear whether total CTC counts are the best measure of their prognostic value. Indeed, rarer CTC subtypes form part of the total CTC count but are poorly isolated by many assays, contributing to the variability of this parameter [53]. A small body of evidence is also emerging that suggests CTC clusters, CTCs undergoing EMT, and other subtypes are prognostic in some early-stage cancers. In a cohort of pre-op early-stage NSCLC patients, the OncoBean device could isolate clusters consisting of up to 200 CTCs, and the detection of more clusters correlated to higher disease stage and worse progression-free survival [82]. In addition, the number of CTCs with hybrid and mesenchymal phenotypes has been associated with poorer prognosis in early-stage prostate [70] and breast cancer [83]. However, even less is known about the features of these subtypes that underpin their prognostic value, besides their presence or some associated genetic aberrations [25, 42]. Thus, assays that can capture common and rare CTC subtypes while enabling single cell analysis will likely help to confirm the features that influence their prognostic value, while achieving higher sensitivity for early-stage cancer.

Devices that evaluate larger volumes of blood could yield higher CTC counts. Besides the discussed limitations of current isolation assays, numerous preanalytical factors influence the type and total number of CTCs that are detected. Even the timing of blood collection seems to influence CTC counts, with a recent study showing that the release of breast CTCs (single and clusters) into the blood is higher at night [84]. Methods that offer a way to continuously capture CTCs over longer periods could thereby improve the total number of CTCs isolated, including rarer subtypes, without the need to take large volumes of blood. For instance, using a peripherally inserted surgical guidewire imbued with anti-EpCAM antibodies for 30 minutes was shown to isolate much larger numbers of CTCs relative to a standard 7.5 mL blood draw, irrespective of disease stage, although there were differences between cancer types (breast and NSCLC) [27, 85]. Aiming to improve on this further, the MagWIRE device could isolate thousands of spiked CTCs in a live porcine model [53]. Advances in this area may enable more effective downstream analysis of CTCs (Figure 2B), as the numbers isolated could be sufficient to partition samples for IF and sequencing, due to the lower risk of losing rarer CTC subpopulations.

Lastly, standardising how results are reported will enable more accurate comparisons between studies, enhancing the ability of meta-analyses to validate the potential of CTC-based assays for early cancer detection and risk stratification. As mentioned, differences in blood volumes, patient characteristics and other preanalytical variables are inconsistently reported. In addition, varying definitions of early-stage disease are used, with some studies simply grouping patients into metastatic or non-metastatic for analysis, without outlining the characteristics of all patients. This lack of standardised reporting makes it difficult to compare the performance of CTC-based technologies. To address these issues, studies should outline the clinical characteristics of all patients, including selection criteria, the stage and grade of disease, and the outcomes of interest. This would allow clearer comparisons between studies, especially for those that include stage III patients in sensitivity and specificity calculations for early-stage cancer.

## ctDNA

### An introduction to ctDNA

ctDNA is a subset of cell-free DNA (cfDNA) fragments that originate from cancer cells and circulate in the blood and other bodily fluids. In healthy people, cfDNA is typically present at low levels (1–10 ng/mL) [86]. However, the release of cfDNA into the blood is influenced by various physiological and pathological processes, including pregnancy, exercise, infection and cancer [87]. The association between cancer and elevated cfDNA levels was first reported in 1977 [88]. Subsequent studies identified cancer-associated genetic alterations in cfDNA from the plasma of cancer patients, such as *KRAS* mutations in pancreatic cancer and *EGFR* mutations in lung cancer. In some cases, these alterations were concordant with those found in the primary tumour, especially for pancreatic and lung cancer [86, 89, 90]. Additional cancer-associated structural and sequence variants have been detected in cfDNA since then, including chromosomal rearrangements, copy number variations and altered methylation patterns [86]. Together, these findings helped to establish ctDNA as a distinct type of cfDNA that contains alterations associated with cancer, providing an early indication of its potential as a cancer biomarker.

The sources of ctDNA and mechanisms of its release into the circulation are still under investigation. In healthy individuals, the majority of cfDNA is thought to be released from haematopoietic cells that undergo apoptosis. This hypothesis is supported by the large proportion of cfDNA that contains WBC-associated methylation patterns [86, 91], as well as its modal size of 166 bp, which corresponds to both the length of DNA within a single nucleosome and internucleosomal cleavage events that characterise apoptosis [86]. In contrast, ctDNA is generally shorter (< 150 bp) [92], with reports of improved ctDNA enrichment from cfDNA fragments sized between 90–150 bp [93]. This shorter size is consistent with ctDNA being passively released from cancer cells that undergo apoptosis, including primary tumour cells, CTCs, and metastatic lesions. Necrosis is also a likely contributor to ctDNA release, although it is associated with the production of larger fragments that can reach sizes in the kilobase range [92]. However, the in vitro rates of apoptosis and necrosis did not correlate to cfDNA release in one study, which instead correlated to the proportion of cells in G1 phase [92, 94]. Moreover, ctDNA has been detected in the supernatant of cultured cancer cells in the absence of cell death [92]. These findings suggest that active mechanisms also play a role in ctDNA release, but as the research in this area has focussed on tumour-derived DNA in EVs, the other potential mechanisms of active release have remained underexplored. In addition, the proportion of ctDNA derived from each mechanism and source remains unclear and needs further investigation, as these factors and how they interact affect the amount and characteristics of the ctDNA available for detection.

As the available isolation and detection technologies have advanced over the last few decades, the potential role of ctDNA in early cancer detection has become more apparent. Across multiple studies, these technologies have revealed that ctDNA can be detected at an early stage in many types of cancer [26, 28], such as lung and colorectal cancer [21, 95]. The genetic alterations in ctDNA also generally correlate with those detected in primary tumours and have prognostic value [26, 86]. Taken together with the emerging evidence and its recent application in assays designed to detect multiple types of cancer, ctDNA could play a key role in future early cancer detection strategies.

### The development of technologies for ctDNA detection and analysis

**Figure 3:**
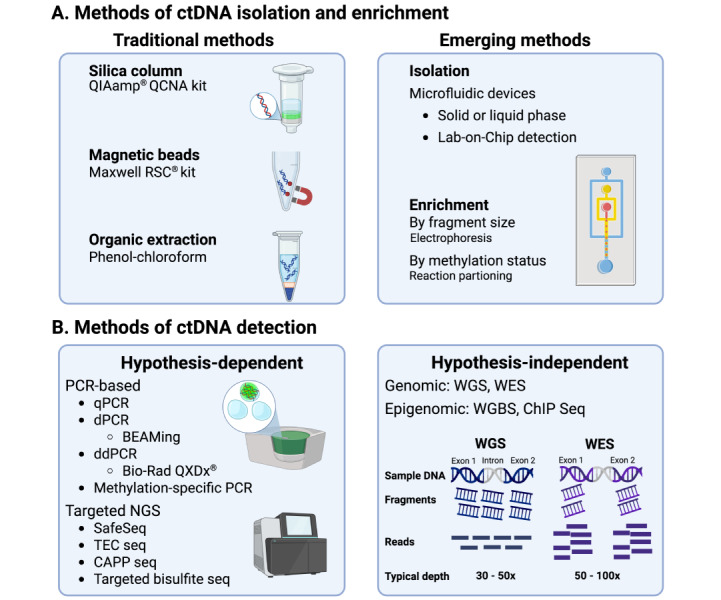
**The methods of isolation and detection of ctDNA.** (**A**) The commonest methods of isolation of ctDNA. Solid phase kits that use silica columns are the most widely used approach for ctDNA isolation, which depend on the adsorption of nucleic acids to the column. Kits using magnetic beads coated in silica or cellulose are also common. In recent years, technological advancements have led to the production of a few microfluidic platforms for ctDNA analysis. Enrichment of ctDNA by selecting for smaller cfDNA fragments has also emerged as a way of improving ctDNA yield and detection; (**B**) the commonest methods of detection of ctDNA. Several methods are used to detect ctDNA. These include sequence-dependent methods, such as PCR and targeted sequencing, as well as sequence-independent methods like whole genome sequencing. Created in BioRender. Thomas, D. (2025) https://BioRender.com/hmo7csj. Created based on references, please see bibliography [87, 104–110]. ctDNA: circulating tumour DNA; BEAMing: beads, emulsion, amplification, and magnetics; CAPP seq: cancer personalised profiling by deep sequencing; ChIP seq: chromatin immunoprecipitation sequencing; PCR: polymerase chain reaction; ddPCR: digital droplet PCR; dPCR: digital PCR; Maxwell RSC: Maxwell rapid sample concentrator; NGS: next generation sequencing; qPCR: quantitative PCR; QIAamp QCNA: QIAamp circulating nucleic acid; TEC seq: targeted error correction sequencing; WES: whole exome sequencing; WGS: whole genome sequencing; WGBS: whole genome bisulfide sequencing

The efficient isolation and accurate detection of ctDNA is challenging due to numerous factors. Although the level of ctDNA in plasma generally correlates to tumour volume and disease stage, it varies considerably across individual patients and cancer types [96]. In late-stage cancer, ctDNA can constitute more than 10% of the available cfDNA in some cases, while in early-stage disease, ctDNA typically forms less than 1% of cfDNA and is often < 0.1% [92, 96]. The half-life of ctDNA in blood is also estimated to be 4–120 minutes, as it is rapidly cleared from the circulation by Kupffer cells in the liver, circulating nucleases, macrophages, and, to a lesser extent, the kidneys [97]. As a result, the traditional polymerase chain reaction (PCR) and Sanger sequencing techniques used in earlier studies lacked the sensitivity needed to accurately detect and characterise ctDNA. Depending on the type of sample and approach, these techniques have detection limits of ~10% and 10–20%, respectively, meaning at least 10% of the cfDNA in a sample should consist of ctDNA for accurate detection [98, 99]. Hence, these methods require impractically large amounts of template and were not sensitive enough to detect mutations among the background noise. Although quantitative PCR (qPCR) has a detection limit of 0.1–1% [100], this improved PCR method was still insufficient for detecting mutations with a low allelic frequency. As summarised in Figure 3, technological advances have led to the development of more effective techniques for ctDNA isolation and detection. In particular, digital PCR and next generation sequencing (NGS) have become widely available [98], allowing these accurate, high-throughput techniques to facilitate the more comprehensive analysis of ctDNA in recent years. Further advances in the approaches to ctDNA isolation and detection will enable its full potential as a tool for early cancer detection to be realised.

Regardless of the approach to isolation, plasma is usually preferred over serum as the starting material. This preference is because serum is prone to contamination from high molecular weight genomic DNA released by lysed WBCs [87, 101, 102], especially if processing is delayed. Thus, before ctDNA extraction, whole blood is typically centrifuged twice to firstly, isolate plasma and, secondly, remove the remaining cells and debris. However, it is still unclear whether blood plasma consistently yields the highest amount and quality of ctDNA relative to serum [87, 100].

Initially, organic extraction techniques using phenol-chloroform were used to isolate ctDNA from blood (Figure 3A). These methods exploit the higher solubility of DNA in the aqueous phase, allowing its separation from the biological molecules trapped in the organic phase and subsequent recovery [103]. However, such approaches are labour-intensive, obtain variable ctDNA yield and quality between extractions, and are more susceptible to contamination with other molecules. As a result, they have largely been superseded by solid-phase extraction techniques, especially those using silica columns or magnetic beads coated with a silica membrane or equivalent (Figure 3A) [87, 100]. These approaches enable the adsorption of DNA to the column or membrane-coated beads under chaotropic conditions, which disrupt hydrogen bonds and encourage salt bridge formation between the phosphate backbone and silica [103]. Due to their ease of use, consistency, and efficiency, silica column and magnetic bead approaches overcame many of the limitations associated with the organic extraction methods for ctDNA isolation.

Both silica column and magnetic bead approaches remain among the most commonly used for ctDNA isolation. Several commercial kits that use silica columns tailored for ctDNA extraction from bodily fluids have been developed (Figure 3B), which have demonstrated high yields and ctDNA quality from plasma in a consistent manner [87, 100]. There have also been reports that silica column kits isolate ctDNA at higher yields than magnetic bead kits, though some studies report no significant difference or the opposite [87, 104]. On the other hand, silica column kits have consistently been shown to have the highest yields for smaller cfDNA fragments, whereas magnetic bead methods appear to isolate both smaller and larger cfDNA without preference [104]. Careful interpretation of such comparisons is vital, as numerous variables between studies are likely to have contributed to these differences beyond the isolation methods themselves. These include the time taken to process the sample, the tubes used, how they are stored, and the methods used to detect the measured ctDNA [105]. It is still unclear if there are truly significant differences between the yield, size or cancer-associated alterations of ctDNA isolated by these methods. Thus, further technological advances will be needed to overcome the potential limitations of current isolation methods, including pre-analytical variables.

Microfluidic technologies are emerging as a potential approach to streamline ctDNA isolation. For example, one study developed a UV/O_3_-activated device that integrated silica binding surfaces into microchips. This device was able to recover between 70–90% of model cfDNA fragments across a diverse range of sizes, with high efficiency for shorter fragments (< 50 bp) [106, 107]. The chip could also be used to detect *KRAS* mutations in plasma samples from lung cancer patients [107]. In addition, another group integrated a microcolumn-based microfluidic device with Sanger sequencing to detect > 70% of *KRAS* and *BRAF* mutations present in the plasma of colorectal cancer patients, including some early-stage cases [108]. Relatively few studies have examined the potential of microfluidic devices in early cancer detection. However, from these studies, it appears that the technology utilised for isolation in the microfluidic devices was sufficient to enable the detection of ctDNA in patient samples using Sanger sequencing, which is generally less accurate than more recently developed methods. Further progress here could enable the integration of ctDNA isolation, enrichment, and detection onto a single platform.

The development of the beads, emulsion, amplification, and magnetics (BEAMing) technique, a form of digital PCR (Figure 3B), marks an important milestone in the evolution of ctDNA detection technologies. This technique works by partitioning ctDNA into an oil-water emulsion, generating millions of individual PCR bubbles. Each bubble contains a magnetic or streptavidin bead coated in primers, which acts as a platform for the reactions to occur. Thus, after amplification, each reaction can be isolated with magnets or the high affinity interaction between streptavidin and biotin, allowing their detection with the fluorescent probes that label the target strand [109]. This technique can detect ctDNA at a fractional abundance of 0.01%, offering significantly higher sensitivity [100]. However, the technical aspects of carrying out BEAMing are impractical for high-throughput detection, as setting up the reactions is laborious, and the number of target strands that can be concurrently detected is limited [98]. Some of these caveats were addressed with digital droplet PCR (Figure 3B). This assay requires fewer technical steps and, because it involves diluting the template to ensure only one strand is present in each bubble, has comparable sensitivity while remaining easy to standardise between labs [110, 111]. On the other hand, NGS techniques have established themselves as the ideal choice for comprehensive sequencing.

NGS is a group of sensitive, high-throughput techniques used to identify sequences of interest in either a hypothesis-driven or hypothesis-independent manner (Figure 3B). For the former approach, the first step involves fragmenting and combining the ctDNA template with adapters [112]. These adapters facilitate the attachment of ctDNA to the interface where sequencing occurs. Depending on the platform or practical needs, they can also act as primers for template amplification and, usually, contain unique identifiers that enable library generation and sample pooling [111, 112]. After adapter ligation, PCR is commonly performed to enrich for sequences of interest before parallel sequencing on the relevant platform. DNA SafeSeq and cancer-personalised profiling by deep sequencing (Figure 3B) are common NGS techniques that use this approach to obtain accurate, high-throughput sequences of targeted genomic regions that frequently acquire the mutations detected in ctDNA [111, 113]. These mostly include mutations in tumour suppressor or oncogenes, with SafeSeq being especially accurate because its unique identifiers allow mutations in both strands to be confirmed, reducing the impact of FP results from PCR or the process of sequencing [111]. In contrast, whole exosome or whole genome sequencing uses a hypothesis-independent approach (Figure 3B). They comprehensively sequence entire regions of interest based on a reference genome, excluding or including introns, respectively. Their lack of focus, however, reduces the overall depth achieved, meaning they are generally more prone to FP results and less effective at detecting mutations with low allelic frequency relative to targeted approaches [112].

Moreover, bisulfite sequencing is another important NGS method. In contrast to the other NGS techniques, this assay facilitates the identification of methylated sequences in the template strand (Figure 3B). By denaturing the template, bisulfite triggers the conversion of unmethylated cytosines into uracil [114]. After PCR, this allows the methylated cytosines to be easily identified by sequencing, as 5-methycytosine will not react with the bisulfite and therefore remain the same. More recently, bisulfite-independent conversion for methylation-based sequencing and digital PCR has also become available for ctDNA detection [114]. Together, these techniques allow the routine molecular profiling of ctDNA, in comparison to the primary tumour.

### The applications of ctDNA in early cancer detection

In one of the earliest studies to assess the utility of ctDNA for cancer detection [115], 55% of 223 localised (stage I/II) cancer patients had detectable ctDNA in their blood samples. To evaluate ctDNA in this study, the mutation status of tumour tissue was first determined. This facilitated the assessment of whether cancer-derived variants in tumour tissue could be detected in plasma as ctDNA. Using a tiered approach, mutations in tumours were either detected with digital PCR, SafeSeq or, if no mutations were found by the panel, whole exome sequencing. ctDNA was then detected by digital PCR or SafeSeq. By integrating different methods, their individual limitations were thereby minimised. Notably, ctDNA was present at detectable levels in 49–78% of patients with early-stage pancreatic (lowest), breast, gastroesophageal, and colorectal cancer (highest). In addition, the study found that higher ctDNA concentrations were associated with poorer 2-year survival. Overall, the results showed that ctDNA was detectable in the plasma of patients with multiple types of early-stage cancer. Findings consistent with this study have been obtained using a variety of methods across different types of cancer [96, 116, 117].

In a similar study, targeted error correction sequencing was applied to detect ctDNA in various cancers [117]. By integrating a hybridisation step before PCR, the ctDNA that contained mutations from the 55-gene panel used in the study was selectively captured from the amplified templates with probes. The enriched ctDNA templates were then redundantly sequenced to achieve higher depth (> 30,000-fold), enhancing the detection of mutations with low allelic frequency while lowering the risk of FP results. For the 200 patients with localised cancer, 62% could be detected with this targeted approach across all tumour types included. For lung, ovarian, and colorectal cancer, the detection rate was 59%, 68%, and 71%, respectively. In addition, high ctDNA levels (mutant allele fraction ≥ 2%) were associated with significantly poorer overall and progression-free survival for the stage I-III colorectal cancer patients in the study. Crucially, this approach did not appear to cause FP results in any of the 44 healthy individuals tested, suggesting that further developments in ctDNA-based technology could enable their application as tests for early-stage cancer in asymptomatic, lower risk patients.

Although these studies were groundbreaking at the time, their results and limitations highlighted the likely challenges of using ctDNA for early cancer detection without further improvements in the technology or in how they are used. In this context, the most relevant issue would be the low sensitivity for early-stage cancer patients. As all the patients were known to have cancer, the reported detection rates and sensitivities would be overestimates. In addition, like most other studies in the field, the controls did not have underlying conditions that needed to be distinguished from cancer, meaning the specificities would have been overestimated as well. Moreover, clonal haematopoiesis of indeterminate potential (CHIP) was not assessed by many earlier studies. This condition is associated with the presence of somatic variants in genes associated with haematological cancers, and its prevalence increases with age (~5% in people aged 60–69) [118]. Given the characteristics of these cohorts, some FP results from this condition would be expected in the tested cancer patients. Indeed, in the second study, 16% of healthy individuals tested positive for such mutations [117], but it is unclear whether cancer patients who only had detectable ctDNA with these mutations were excluded or included as positive events for the reported detection rates.

More recently, a methylation-based ctDNA assay, PanSeer, was shown to improve on the performance of these earlier studies [119]. In this large longitudinal study, patients provided blood samples and were monitored to assess whether they would develop five types of cancer. Once they were diagnosed, the blood samples were then retrospectively analysed with targeted bisulfite NGS, covering a panel of 595 genomic regions. The obtained methylation profiles were then assigned a score with a logistic regression classifier to predict a diagnosis of cancer. The classifier had a sensitivity and specificity of 88% and 96%, respectively, for the 113 post-diagnosis cancer patients and 207 healthy controls. Remarkably, a similar distribution of scores was obtained for both early- and late-stage patients, suggesting the test was equally sensitive for early-stage disease as well. The sensitivity was as high as 96% for lung cancer. In addition, the test was positive in 96% of individuals who were asymptomatic at the point of recruitment and went on to develop cancer 4 years later, with a sensitivity of 91% for oesophageal cancer, regardless of stage. This study was the first to demonstrate that ctDNA-based assays could potentially detect or predict the onset of multiple types of early-stage cancer with relatively high sensitivity, including for asymptomatic patients. Moreover, as methylation-based tests detect tumour-specific methylation patterns, they are unlikely to be influenced by CHIP, enhancing specificity. However, the study needs independent validation, and the cohort was at higher risk for the assessed cancers. Also, in other methylation-based studies, the sensitivities in detecting early-stage cancers are still limited, despite the presence of the tested methylation patterns in tumours [120]. This suggests further confirmation of the most representative methylation patterns for each type of early-stage cancer could still be needed.

### Future directions of ctDNA in early cancer detection

In cancer screening, a multi-cancer early detection (MCED) test has many advantages over those aiming to identify a single type of cancer, therefore, MCED is the future direction for early cancer detection. Over the last few years, ctDNA-based technologies have been applied as MCED tests, such as Galleri, another methylation-based targeted NGS test. Galleri aims to detect up to 50 different cancers at earlier stages. In the SYMPLIFY-Oxford study that used Galleri to test a symptomatic, high-risk cohort, some promising initial results were obtained [121]. Although the sensitivity was highest in late-stage cancers and the number of early-stage cases was low, the test achieved high sensitivity for pancreatic (91.7%) and upper GI cancers (95.5%) across all stages. In addition, the overall specificity of the test for the assessed cancers was 98.4%, and it could accurately predict the site of origin with an accuracy of 84.8%. To date, pancreatic cancer does not have a screening test and, similarly to upper GI cancers, is usually diagnosed late. While endoscopy is available as a screening and surveillance tool for individuals at high risk of oesophageal cancer, and recent evidence supports the use of Cytosponge for these purposes [122], the latter is only available in England and Wales as part of a clinical trial. Methylation-based ctDNA assays like Galleri could therefore potentially help these patients achieve an earlier diagnosis. In agreement with these findings, the results of the preceding Circulating Cell-free Genome Atlas (CCGA) validation study achieved a broadly similar performance using Galleri [123]. However, the results from both studies highlighted the overall low sensitivity of Galleri for detecting early-stage cancer (SYMPLIFY: 24.2% for stage I; and CCGA: 16.8% for stage I). The prospective PATHFINDER study on an asymptomatic cohort also reported a FP rate of 62% using the test [124]. Together, these issues highlight the likely need for further improvements in the technology of such assays or how they are applied. Further evaluation of the test for MCED is underway in the NHS-Galleri RCT on a large cohort of asymptomatic patients, and its results, due in 2026, are highly anticipated.

Evaluating the broader impact of these tests not only requires the careful assessment of their detection performance but also the outcome measures used. For the ongoing NHS-Galleri RCT (NCT05611632), the primary outcome is the absolute numbers of stage III and IV cancers, with one of the secondary endpoints being cancer-specific mortality. In addition, the trial aims to achieve a 30% reduction in stage IV cases. The choice of outcome measures is key because some of these could be achieved without necessarily improving outcomes that matter to patients, such as all-cause or cancer-specific mortality. For instance, even if the absolute numbers of late-stage disease decrease, they alone do not confirm whether patients diagnosed earlier will achieve a benefit of reduced mortality. In addition, an RCT of an average risk population in the UK recently found that although multimodal screening reduced the incidence of late-stage ovarian cancer by 10.2%, the 39.2% rise in early-stage diagnoses was not followed by a significant reduction in mortality [125, 126]. Although a recent meta-analysis showed that reductions in the incidence of late-stage disease strongly correlate to a reduction in ovarian cancer-specific mortality, only a weak correlation was observed for colorectal and prostate cancer [127]. Together, these findings suggest that a reduction in the incidence of late-stage disease, and any subsequent stage shift, may not be an appropriate surrogate measure for cancer-specific mortality, depending on the type of cancer. Assessing all-cause mortality could be the ideal outcome measure for MCED tests, as an overall reduction in this parameter across the cancers they detect earlier would provide clearer evidence of a mortality benefit and cost effectiveness.

Researching the dynamics of ctDNA may improve the ability to diagnose cancer earlier with ctDNA-based assays. The half-life of ctDNA is thought to be in the minutes to hours range, but the timing of ctDNA release is poorly characterized [87, 97]. Similarly to other CTBs, standardising protocols to minimise preanalytical issues will also be crucial for further improvements in the performance and validation of ctDNA-based tests [128]. For instance, it is generally recommended to avoid EDTA-containing tubes and instead use Streck cell-free tubes or an equivalent. These have been shown to improve the stability of cfDNA and reduce haemolysis, preventing the dilution of ctDNA by genomic DNA or DNA enriched with alterations that are associated with CHIP [128, 129]. In addition, sequencing peripheral blood mononuclear cells could be used as an internal control to avoid FPs from mutations associated with CHIP [128, 129], but CTCs should be removed first. Furthermore, recent studies have focussed on shorter ctDNA fragments to enrich for somatic variants and copy number alterations [92, 93, 128]. While there is no clear evidence to show that the current extraction processes inadvertently reduce the amount of long cfDNA fragments obtained in favour of shorter ones [129], these studies generally did not examine fragments larger than ~1,500 bp. This lack of assessment is important because some early studies have identified much larger cfDNA fragments in cancer patients, which might be consistent with higher rates of necrosis [92, 130]. Increased rates of necrosis are associated with poorer prognosis in various cancers [92, 131]. Thus, ensuring large fragments are captured and applying more recent methods of analysis, such as methylation-based profiling, could yield new insights related to detection and prognosis for early-stage cancer patients.

Integrating multiple approaches to detect ctDNA may improve the accuracy of ctDNA-based assays for early cancer detection. One large prospective study recently applied a multimodal approach to detect ctDNA in an average-risk cohort for bowel cancer [132]. The assay was designed to use targeted NGS to identify ctDNA with both specific methylation patterns and mutations associated with colorectal cancer. Combined further with fragmentation patterns, these parameters were used to generate a score via a logistic regression model, which predicts the likelihood of colorectal cancer. Strikingly, the test had a sensitivity of 87.5% for stage I-III disease and had a specificity of 89.9% for a negative colonoscopy. The test also had a sensitivity of 13% for detecting advanced precancerous adenomas. Thus, integrating different approaches of analysis for ctDNA could enhance their detection performance.

## Emerging novel circulating biomarkers for early detection

### Circulating EVs

EVs have emerged as a promising class of CTBs for early cancer detection. EVs are a diverse group of lipid bilayer-enclosed nano-sized particles generated by all cell types. Produced by the endocytic pathway or outward budding of the cellular membrane, the most well-studied EV subtypes are exosomes and microvesicles, which serve as carriers of proteins, DNA, and other biologically active molecules [133]. In the past, EVs were primarily considered as waste transporters. However, recent work has resulted in the recognition of EVs as mediators of intercellular trafficking and communication, which can enhance tumour growth, promote resistance to treatment and even manipulate normal cells to further these processes [134].

Cancer-secreted EVs play a key role in how cancer cells communicate with and shape their environment [135, 136], influencing multiple steps in metastasis. Both in vitro and in vivo studies have demonstrated the correlation between increasing concentrations of EVs and tumour invasiveness [135, 137]. Circulating EVs have also been shown to systemically suppress anti-tumuoral immunity, facilitating cancer cell survival [138–142]. EVs strongly influence the ability of CTCs to form metastases by affecting multiple steps in the cascade [136, 140–143]. Particularly, they help to prepare the pre-metastatic niche for the survival and outgrowth of cancer cells, after CTCs arrive in their new microenvironment [135, 140–143], therefore, EVs can be obtained from plasma samples for analysis.

Methods for isolating and detecting EVs have significantly improved over the years. Ultracentrifugation and size-exclusion chromatography are traditional approaches that remain widely used for EV isolation, but other methods are gaining traction. Recent efforts have shifted towards immunoaffinity-based assays and integrative microfluidic devices like those applied for CTC capture, although other novel techniques are also being explored [144]. Due to the distinct morphological features of EVs and the diverse cargo they contain, a wide range of methods can be used to detect them. These include but are not limited to: electron microscopy, flow cytometry, immunoassays, digital droplet PCR, and sequencing [144]. Despite the challenges involved with obtaining pure EVs from plasma or other bodily fluids, they are particularly stable and, due to the variety of their cargo, facilitate more complex profiling of the tumours from which they are released. Thus, EVs could potentially offer some advantages over CTCs and ctDNA-based methods of cancer detection.

Some recent studies have illustrated the potential utility of EV-based assays for early cancer detection. In a case-control study, EVs were isolated from the plasma of cancer patients using a novel integrative device and then detected by a multiplex immunoassay based on 42 EV-associated proteins [145]. Strikingly, the platform could detect stage I pancreatic (96%) and ovarian cancer (74%) at high sensitivity, with a specificity of 99.5% across all cancers tested (AUC = 0.95). In addition, another study developed a model to classify early-stage lung cancer based on the differential expression of long RNAs contained in EVs [146]. This approach detected early-stage lung adenocarcinoma at a sensitivity and specificity of 93.75% and 85.71%, distinguishing it from pulmonary infection and benign lung nodules (AUC = 0.918). Together, these results highlight the potential of tumour-derived EVs in the early detection of ‘less survivable’ cancers.

### Circulating protein biomarkers

Although proteins were the first circulating biomarkers explored for clinical use, they were less investigated over the last two decades compared to CTCs and ctDNA. The traditional focus of circulating protein biomarker research was to find individual tissue-specific proteins that could distinguish cancer patients from the healthy [147]. However, the classic serum biomarkers were ineffective at achieving this for early-stage cancer, as they lack the required sensitivity and specificity when used alone [23, 147]. Due to the advances in antibody and mass spectrometry (MS) technology, novel protein combinations and proteomic approaches have been gaining attention. Hence, recent research has focussed on hypothesis-driven multiplex assays to simultaneously detect a panel of known proteins, as well as high-throughput, data-independent MS techniques for new biomarker discovery [147].

The antibodies used in some multiplex assays have improved considerably in recent years. One class of these improved antibodies are applied in proximity extension assays. This technology uses antibody pairs, with each antibody being conjugated to a unique oligonucleotide that is complementary to the other. Thus, when a pair of antibodies bind to the antigen of interest and are in close proximity, their unique identifiers ligate, allowing their detection by PCR or NGS [148]. This method, therefore, utilises the specificity of antibodies with the sensitivity of nucleotides to identify targets of interest, even if they are expressed at relatively low levels in serum.

One recent study showed that a multiplex assay using proximity-extension antibodies could detect ~3,000 low and high-abundance serum proteins across 440 patients [149]. After using an ML algorithm to generate classifiers, the serum proteins were then narrowed down into 10 candidate biomarkers. The results showed that by using these classifiers, the 10 candidate proteins had an overall 90% sensitivity in men and 85% in women at 99% specificity across a range of common cancers, with an AUC of ~0.98 for both sexes. In addition, the sensitivities were similar for early-stage cancer, as the test identified 93% of males and 84% of females with stage I cancer. Although the number of stage I patients included was small and the findings have not been independently replicated yet, the study suggests that protein panels, aiming to account for the complexity of cancer, could be a feasible approach for early cancer detection.

### Synthetic biomarkers

Synthetic biomarkers are an emerging class of biomarkers designed to bypass some of the challenges associated with endogenous biomarker detection. In early-stage cancer, CTCs and ctDNA are generally present at lower levels compared to more advanced disease. Synthetic biomarkers aim to overcome this obstacle by producing measurable de novo signals through their interactions with cancer-associated factors. Rather than directly detect endogenous biomarkers, they intend to amplify cancer-specific signals. In one study, a panel of 14 nanosensors was developed for the detection of lung cancer [150]. These nanosensors were designed to be cleaved by specific proteases in the tumour microenvironment, such as matrix metalloproteases. Cleavage events would then expose the unique barcode formed by stable isotope amino acids, which could then be detected with imaging or other techniques. The nanosensors were further developed into an aerosolised version for testing on mutant *Kras^G12D^-Trp53^fl/fl^* KP mice, designed to mimic the common mutations found in lung adenocarcinoma. The sensitivity and specificity of detecting lung tumours using nanosensors visible on micro-CT were 81% and 100%. Excitingly, the cleaved nanosensors were excreted in urine and detectable across a wide dynamic range with liquid chromatography-MS. Although these biomarkers are certainly in the preclinical stages, such synthetic biomarkers could be a cost-effective way of screening for lung cancer if lateral flow devices were developed for their detection.

## Conclusions

The technologies available to isolate and analyse CTCs, ctDNA, and other CTBs have exploded in the last two decades. Promising results from some studies are already demonstrating the strengths that CTCs, ctDNA and tumour-associated EVs hold in comparison to more widely available methods of detection. These novel biomarkers generally have high specificity, are easily tested, and provide a wealth of prognostic and actionable information. In addition, further research into their roles within the pathogenesis of cancer could improve our understanding of this heterogeneous disease, reveal even more effective versions of these biomarkers and aid the development of new treatments.

However, the current sensitivity and specificity of these novel CTBs risks considerable FP rates. Even if the overall sensitivity and specificity of a given assay are both 95% and the prevalence of the cancer in question is 500 per 100,000, around 500 people would still experience a FP result in a population of 10,000 [151]. As the performance of these tests varies considerably by cancer prevalence, stage and type, especially in average-risk populations, this issue could be a major challenge for CTBs assays to overcome, including MCED assays. On the other hand, repeated testing with CTBs that have higher specificity, while combining them with the available sensitive methods, could at least partly address this issue.

The available evidence clearly points to the benefits these tests may offer high-risk patients. ctDNA and CTCs showed consistently better performance and independent prognostic value for multiple early-stage cancers in cohorts that included patients who were symptomatic or had risk factors. Given the Faster Diagnosis Standard recently introduced by the UK government [152], these CTBs could be an excellent way to help differentiate patients who need urgent testing. In the UK, most cancer diagnoses are achieved by urgent primary care referral of patients suspected to have cancer [153]. These patients are more likely to have symptoms or risk factors, increasing the positive-predictive value. In addition, patients who might not meet the criteria for an urgent referral, such as those with non-specific symptoms, could benefit from these tests, especially with further risk stratification based on their primary care records [154].

In addition, the current evidence highlights an unmet need for the effective detection of less survivable cancers, which CTB assays could help to address. As mentioned, almost half of all cancers were diagnosed at stage III/IV in England before the COVID-19 pandemic [12]. Many of the diagnoses at these advanced stages are ‘less survivable’ cancers, such as lung, pancreatic, hepatic, oesophageal, gastric, brain, and ovarian cancer [155, 156]. The former six cancers alone are estimated to account for over 40% of cancer deaths in both the UK [156] and the US [6]. Moreover, these cancers broadly lack effective detection or screening methods, indicating that strategies to detect these and other cancers at earlier, more treatable stages could significantly reduce the overall burden of cancer-related mortality. Although the number of studies on CTBs was more limited for the less survivable cancers, we provide evidence that shows their emerging potential for the early detection of such cancers, representing an important avenue for future research.

As cancer is a dynamic, constantly evolving system that exhibits substantial interpatient variability [147], combining different classes of CTBs is likely to be the best approach for future early cancer detection strategies. The CancerSEEK test is one of the notable early examples [157], which combined 8 serum proteins, including CEA and CA-125, with a 61-amplicon panel of common somatic variants for ctDNA detection. Although the test has a median sensitivity of 43% for stage I and 78% for stage II cancers, it could detect stage I liver cancer with 100% sensitivity. The test also localised the site of origin with a median sensitivity of 63%. Recent studies have shown that the combination of CTCs and ctDNA was more efficient for cancer detection than their individual use [158, 159]. For instance, a small cohort study that combined CTCs with ctDNA detected stage I-II NSCLC patients at a sensitivity of 91.2% [160], which was equally high for other lung cancer subtypes. Further work to examine the various combinations of novel and established biomarkers will likely yield improved detection performance by integrating their advantages and neutralising singular weaknesses.

## Abbreviations

AUC: area under the receiving operator characteristics curve
BEAMing: beads, emulsion, amplification, and magnetics
CA-125: cancer antigen-125
CD45: cluster of differentiation 45
CEA: carcinoembryonic antigen
cfDNA: cell-free DNA
CHIP: clonal haematopoiesis of indeterminate potential
CNN: conventional neural network
CTBs: circulating tumour biomarkers
CTCs: circulating tumour cells
ctDNA: circulating tumour DNA
DTCs: disseminated tumour cells
EMT: epithelial-mesenchymal transition
EpCAM: epithelial cell adhesion molecule
EVs: extracellular vesicles
FNs: false-negatives
FP: false-positive
IF: immunofluorescence
MCED: multi-cancer early detection
ML: machine learning
MS: mass spectrometry
NGS: next generation sequencing
NSCLC: non-small cell lung cancer
PanCK: pancytokeratin
PCR: polymerase chain reaction
PSA: prostate-specific antigen
RBC: red blood cell
RCT: randomised-controlled trial
SVM: support vector machine
WBC: white blood cell

## Declarations

### Copyright

© The Author(s) 2025.
